# Supplementary material for: How to design a pre-specified statistical analysis approach to limit p-hacking in clinical trials: the Pre-SPEC framework
Source: BMC Med. 2020 Sep 7;18:253. doi: 10.1186/s12916-020-01706-7 (PMC7487509; doi:10.1186/s12916-020-01706-7)
Supplement: Supplementary file 1 — Additional file 1. [file 12916_2020_1706_MOESM1_ESM.docx]

**Additional file 1: Table S1**

**Table S1 – Comparison of the Pre-SPEC framework with the SPIRIT and ICH-E9 guidelines**

| **Pre-SPEC framework** | **SPIRIT** | **ICH-E9** | **Comment** |
| --- | --- | --- | --- |
| Pre-specify before recruitment to the trial begins | - “The planned methods of statistical analysis should be fully described in the protocol” - “The protocol should indicate explicitly each intended analysis comparing study groups. An unambiguous, complete, and transparent description of statistical methods facilitates execution, replication, critical appraisal, and the ability to track any changes from the original pre-specified methods.” | - “For each clinical trial contributing to a marketing application, all important details of its design and conduct and the principal features of its proposed statistical analysis should be clearly specified in a protocol written before the trial begins.” (p5) - “When designing a clinical trial the principal features of the eventual statistical analysis of the data should be described in the statistical section of the protocol. This section should include all the principal features of the proposed confirmatory analysis of the primary variable(s) and the way in which anticipated analysis problems will be handled.” p23-24 | Both SPIRIT and ICH-E9 state the planned statistical analysis approach should be pre-specified in the protocol. ICH-E9 explicitly states this should be done before the trial begins; SPIRIT does not state this explicitly, but it is implied given that the first version of the protocol must be completed before the trial begins. |
| Specify a single primary analysis strategy. | - “Results for the primary outcome can be substantially affected by the choice of analysis methods. When investigators apply more than one analysis strategy for a specified primary outcome, there is potential for inappropriate selective reporting of the most interesting result. The protocol should prespecify the main (“primary”) analysis of the primary outcome…” - “When both unadjusted and adjusted analyses are intended, the main analysis should be identified (Item 20a).” | - “The primary analysis of the primary variable should be clearly distinguished from supporting analyses of the primary or secondary variables.” p28 | Both SPIRIT and ICH-E9 state explicitly that a single main analysis strategy should be identified. |
| Plan all aspects of the analysis (including analysis population, statistical model, covariates, and handling of missing data) | - “The protocol should prespecify the main (“primary”) analysis of the primary outcome (Item 12), including the analysis methods to be used for statistical comparisons (Items 20a and 20b); precisely which trial participants will be included (Item 20c); and how missing data will be handled (Item 20c).” - “It is important that trial investigators indicate in the protocol if there is an intention to perform or consider adjusted analyses, explicitly specifying any variables for adjustment and how continuous variables will be handled.” - “Protocols should explicitly describe which participants will be included in the main analyses (eg, all randomised participants, regardless of protocol adherence) and define the study group in which they will be analysed (eg, as randomised).” - “The protocol should also state how missing data will be handled in the analysis and detail any planned methods to impute (estimate) missing outcome data, including which variables will be used in the imputation process (if applicable).” - “Finally, different trial designs dictate the most appropriate analysis plan and any additional relevant information that should be included in the protocol. For example, cluster, factorial, crossover, and within-person randomised trials require specified statistical considerations, such as how clustering will be handled in a cluster randomised trial.” | - “The set of subjects whose data are to be included in the main analyses should be defined in the statistical section of the protocol.” p24 - “The decision to transform key variables prior to analysis is best made during the design of the trial on the basis of similar data from earlier clinical trials. Transformations (e.g. square root, logarithm) should be specified in the protocol and a rationale provided, especially for the primary variable(s).” p27 - “The statistical section of the protocol should specify the hypotheses that are to be tested and/or the treatment effects which are to be estimated in order to satisfy the primary objectives of the trial. The statistical methods to be used to accomplish these tasks should be described for the primary (and preferably the secondary) variables, and the underlying statistical model should be made clear. Estimates of treatment effects should be accompanied by confidence intervals, whenever possible, and the way in which these will be calculated should be identified. A description should be given of any intentions to use baseline data to improve precision or to adjust estimates for potential baseline differences, for example by means of analysis of covariance.” p27 - “All effects to be fitted in the analysis (for example in analysis of variance models) should be fully specified… . The same considerations apply to the set of covariates fitted in an analysis of covariance.“ p28 - “The primary variable(s) is often systematically related to other influences apart from treatment. For example, there may be relationships to covariates such as age and sex, or there may be differences between specific subgroups of subjects such as those treated at the different centres of a multicentre trial. In some instances an adjustment for the influence of covariates or for subgroup effects is an integral part of the planned analysis and hence should be set out in the protocol. Pre-trial deliberations should identify those covariates and factors expected to have an important influence on the primary variable(s), and should consider how to account for these in the analysis in order to improve precision and to compensate for any lack of balance between treatment groups.” p28 | SPIRIT explicitly states that the analysis population, analysis model, covariates, handling of missing data, and any other relevant aspects should be specified. ICH-E9 explicitly states that the analysis population, statistical model, covariates, and use of transformations for key variables should be specified. |
| Enough detail should be provided so that a third party could independently perform the analysis | - “It is important that trial investigators indicate in the protocol if there is an intention to perform or consider adjusted analyses, explicitly specifying any variables for adjustment and how continuous variables will be handled.” - “Protocols should explicitly describe which participants will be included in the main analyses (eg, all randomised participants, regardless of protocol adherence) and define the study group in which they will be analysed (eg, as randomised).” - “The ambiguous use of labels such as “intention to treat” or “per protocol” should be avoided unless they are fully defined in the protocol. … Other ambiguous labels such as “modified intention to treat” are also variably defined from one trial to another.” - “The protocol should also state how missing data will be handled in the analysis and detail any planned methods to impute (estimate) missing outcome data, including which variables will be used in the imputation process (if applicable).” | - “The decision to transform key variables prior to analysis is best made during the design of the trial on the basis of similar data from earlier clinical trials. Transformations (e.g. square root, logarithm) should be specified in the protocol and a rationale provided, especially for the primary variable(s).” p27 - “The statistical section of the protocol should specify the hypotheses that are to be tested and/or the treatment effects which are to be estimated in order to satisfy the primary objectives of the trial. The statistical methods to be used to accomplish these tasks should be described for the primary (and preferably the secondary) variables, and the underlying statistical model should be made clear. Estimates of treatment effects should be accompanied by confidence intervals, whenever possible, and the way in which these will be calculated should be identified. A description should be given of any intentions to use baseline data to improve precision or to adjust estimates for potential baseline differences, for example by means of analysis of covariance.” p27 - “All effects to be fitted in the analysis (for example in analysis of variance models) should be fully specified… . The same considerations apply to the set of covariates fitted in an analysis of covariance.“ p28 - “The primary variable(s) is often systematically related to other influences apart from treatment. For example, there may be relationships to covariates such as age and sex, or there may be differences between specific subgroups of subjects such as those treated at the different centres of a multicentre trial. In some instances an adjustment for the influence of covariates or for subgroup effects is an integral part of the planned analysis and hence should be set out in the protocol. Pre-trial deliberations should identify those covariates and factors expected to have an important influence on the primary variable(s), and should consider how to account for these in the analysis in order to improve precision and to compensate for any lack of balance between treatment groups.” p28 | Both SPIRIT and ICH-E9 state that certain aspects of the analysis should be explicitly or fully described (e.g. analysis population, covariates, handling of missing data, etc). |
| Adaptive analysis strategies should use deterministic decision rules | - “It is important that trial investigators indicate in the protocol if there is an intention to perform or consider adjusted analyses… . It may not always be clear, in advance, which variables will be important for adjustment. In such situations, the objective criteria to be used to select variables should be prespecified.” | - “The particular statistical model chosen should reflect the current state of medical and statistical knowledge about the variables to be analysed as well as the statistical design of the trial. All effects to be fitted in the analysis (for example in analysis of variance models) should be fully specified, and the manner, if any, in which this set of effects might be modified in response to preliminary results should be explained.” p28 | SPIRIT advocates objective decision rules in a single specific instance (if covariates are to be chosen based on trial data). ICH-E9 states that the way the analysis might be modified in response to preliminary results should be specified. To the extent that adaptive analysis strategies are mentioned, both imply that pre-specified deterministic decision rules should be used. |
